# Cytologic Analysis of Epstein-Barr Virus-Associated T/Natural Killer-Cell Lymphoproliferative Diseases

**DOI:** 10.3389/fped.2018.00327

**Published:** 2018-11-16

**Authors:** Akihiro Yachie

**Affiliations:** Department of Pediatrics, School of Medicine, Graduate School of Medical Sciences, Kanazawa University, Kanazawa, Japan

**Keywords:** epstein-barr virus, EBV-associated hemophagocytic lymphohistiocytosis, chronic active EBV infection, hydroa vacciniforme, hypersensitivity to mosquito bite, flowcytometry

## Abstract

Rapid, precise diagnosis of Epstein-Barr virus-associated T lymphocyte or natural killer cell lymphoproliferative diseases is clinically important to prevent disease progression and avoid fatal outcomes for patients. In addition to detecting increased copy numbers of Epstein-Barr virus, identification of the lymphocyte subpopulation targeted by the virus infection is crucial to reaching the final diagnosis. However, these procedures are laborious and require large amounts of sample. In contrast, flowcytometric analysis may provide crucial information for initial screening of diseases using only small amounts of sample and involves little labor. In addition to the increase of a particular subpopulation, selective HLA-DR expression indicates selective activation and expansion of a virus-infected clone. Presence of a characteristic HLA-DR^high^ CD5^dim/negative^ fraction within CD8^+^ T lymphocytes indicates a possible diagnosis of Epstein-Barr virus-associateds hemophagocytic lymphohistiocytosis. One should note, however, that cases with familial hemophagocytic lymphohistiocytosis may exhibit a similar abnormal fraction within CD8^+^ T lymphocytes. These T cells are oligoclonally expanded reactive T cells expanding in response to B cells infected with Epstein-Barr virus.

## Introduction

Epstein-Barr virus (EBV) is one of the most ubiquitous infectious viruses, with >90% of the human beings infected worldwide (1, 2). Primary EBV infection occasionally results in infectious mononucleosis in children and young adults. In the majority of individuals infected with EBV, latent infection is established for life without any specific clinical manifestation. In primary EBV infection, B cells are the cellular targets of EBV and latent EBV infection persists for life in B cells, as well as in nasopharyngeal cells. For this reason, these cells play an important role as a reservoir for EBV (3). In hosts with normal immune function, EBV latently infects B cells without any pathological disorders, but EBV is also known to play significant roles in the pathogenesis of various non-hematological and hematological diseases. EBV rarely causes two clinically distinct and unusual infections: the acute form of fulminant life-threatening diseases with hemophagocytic lymphohistiocytosis (HLH); and chronic persistent infection associated with systemic organ involvement and malignant transformation at the late stage (4, 5). These two diseases are clinically defined as EBV-associated HLH (EBV-HLH) and chronic active EBV infection (CAEBV), respecti*v*ely. In these two diseases, ectopic EBV infection in lymphocyte subpopulations other than B lymphocyte has been demonstrated and plays significant roles in the pathogenesis of these diseases (6). As the major targets of EBV infection are T cells and natural killer (NK) cells, these pathologies are collectively called EBV-associated T/NK-cell lymphoproliferative diseases (LPD). Although asymptomatic primary EBV infection or acute infectious mononucleosis usually require no specific treatment, early diagnosis, and therapeutic interventions are critical for patients with EBV-HLH or CAEBV. In this article, I briefly review the clinical characteristics of the two important EBV-associated T/NK-cell LPDs. Next, the particular utility of cytologic analysis in the diagnosis of these pathologies is frequently overlooked, but the clinically significant illnesses are discussed.

## EBV-associated hemophagocytic lymphohistiocytosis

HLH is an acute systemic inflammatory illness characterized by macrophage activation, hemophagocytosis in the bone marrow, pancytopenia, and hepatosplenomegaly, all of which reflect intense inflammatory cytokine production *in vivo* (7). HLH has multiple primary causes, including infections, collagen vascular diseases, malignancies and some metabolic diseases. Primary immunodeficiency diseases affecting NK function and killer T-cell functions are also the targets of HLH. EBV is the most common triggering agent of HLH in Japan, particularly among children 1–15 years old (8). Because immunochemotherapy with etoposide and corticosteroids can be lifesaving for patients with EBV-HLH, early correct diagnosis is of paramount importance (9, 10). Left unrecognized, these patients may experience rapidly progressive cytokinemia and deterioration of multiple organ functions. Although the HLH-2004 protocol has been shown to be helpful in establishing the diagnosis of HLH, some findings in these criteria only occur late in the disease course, and therapeutic intervention is thus often delayed if clinicians wait until the criteria are satisfied (11, 12). Data on valuable diagnostic parameters, including EBV copy numbers, profiles of inflammatory cytokines, NK cell function, and mutation analysis of the genes related to genetic HLH, are only available from specialized laboratories. In addition, it is difficult to distinguish EBV-HLH from IM by serological tests for EBV and routine immunophenotypic analysis of lymphocyte subsets. Patients with IM sometimes exhibit marked T cell activation and cytokine production to regulate EBV-infected B cells. Therefore, infectious mononucleosis may share some typical clinical features with EBV-HLH, such as cytopenia, hypercytokinemia, and hemophagocytosis, even though IM involves a benign self-limited episode and usually does not require any specific treatment. Diagnosing EBV-HLH in the early stage thus remains difficult.

In acute infectious mononucleosis, B cells are the targets of EBV infection and T cells are activated to control the expansion of these EBV-infected B cells. In contrast, EBV ectopically infects CD8^+^ T cells without producing a sufficient number of EBV-specific cytotoxic T cells in EBV-HLH (6). For this reason, therapeutic intervention differs critically for these two distinct categories of EBV-associated lymphoproliferation. Controlling hypercytokinemia is a sufficient goal in acute infectious mononucleosis. In contrast, control of abnormal expansion of EBV-infected CD8^+^ T clones is mandatory in addition to cytokine regulation in EBV-HLH. Finding targets of EBV infection during the acute episode of EBV-HLH is technically difficult when the peripheral lymphocyte count is very limited and the patients are generally young. A novel rapid and easy diagnostic approach to detect clonal expansion of EBV-infected CD8^+^ T cells is thus highly desirable.

## Chronic active EBV infection

CAEBV is characterized by prolonged or intermittent IM-like symptoms, such as fever, general malaise, liver dysfunction, lymph node swelling, and hepatosplenomegaly. In CAEBV, EBV infects lymphocytes other than B lymphocytes (13). Proof of ectopic EBV infection is very important in the precise diagnosis of CAEBV. We do not know at present if common pathogenetic mechanisms underlie the onset of each CAEBV case. However, it is very important that these cases share common clinical features, such as ectopic infection by EBV of a single clone of NK cells or T lymphocytes, and appearance during the course of illness of an acute episode of HLH or malignant transformation of the EBV-infected cells at later stages. Diagnostic criteria for CAEBV include the following according to the guideline proposed in 2005 (14):

- Persistent or recurrent IM-like symptom;
- Unusual pattern of anti-EBV antibodies with raised anti-VCA and anti-EA, and/or detection of increased EBV genomes in affected tissues, including peripheral blood; and
- Chronic illness that cannot be explained by other known disease processes at diagnosis.

The cardinal features of the above criteria are the detection of EBV in cells other than B lymphocytes (NK cells, CD4^+^ T cells, CD8^+^ T cells or TCRγδ T cells), and confirmation of increased EBV genome in the peripheral blood or tissue specimens. CAEBV consists of several different clinical phenotypes (15). Distinct clinical profiles for each phenotype seem to reflect the differences in the targets of EBV infection in each disease. Two well-characterized CAEBV and related illnesses with distinct skin manifestations are hypersensitivity to mosquito bite (HMB) and hydroa vacciniforme (HV).

HMB is known for its distinct clinical features, including intense local skin reaction to mosquito bite, which includes early inflammatory vesicle formation soon after mosquito bites, followed by development of a large erythematous lesion with central necrosis or crust (16). The tissue damage is so intense that the vesicles are often associated with intravesicular hemorrhage. Dermal inflammation is generally very deep, resulting in ulceration and necrosis with thick crust and scar formation (17). Patients often show old scars from mosquito bites of the previous seasons. The severe skin lesions are associated with systemic symptoms, including fever and general malaise. Liver dysfunction is generally observed. For severe cases in later years, patients with HMB may show signs of HLH and hypercytokinemia. Virtually all patients with HMB show high titers of specific immunoglobulin (Ig) E against mosquito antigens and have antigen-specific CD4^+^ T cells. Peripheral blood basophils and T lymphocytes from patients therefore show positive reactions in response to mosquito antigens *in vitro* (18, 19).

Because the clinical characteristics of HMB are relatively easy to recognize, diagnosis can be made early after onset, once the physician can recall this particular illness from the typical symptoms. However, some conditions show a similar clinical presentation after mosquito bites. Some patients who have specific IgE antibody to mosquito antigen may show immediate, intense skin reaction after mosquito bites. These skin lesions do not persist for long and the lesion will heal without forming local necrosis or scar formation (20). The patients never present with fever, malaise, or liver dysfunction. Another subset of patients who show intense reaction to mosquito bites present with systemic symptoms including fever and liver dysfunction. Again, the skin lesions are not severe and go away within several days without scar formation. Clinical diagnosis of HMB is thus not very difficult, but requires demonstration of an increase in EBV copy number in peripheral blood and ectopic EBV infection of NK cells, and rarely other non-B lymphocytes. Flowcytometric (FCM) analysis offers rapid screening to detect increased, activated NK cells in these patients.

HV is another unique EBV-associated LPD, in which the patient shows characteristic skin lesions on ultraviolet-exposed areas, including the ear lobes, dorsal surfaces of the hands, and cheeks. HV can be divided into a benign classic type and more aggressive systemic-type illnesses (21, 22). The skin lesions often become worse in seasons with increased UV exposure, typically May through September. Some patients show acute aggravation of skin lesions after going skiing in winter because of the intense reflection of UV from the snow-covered ski area. Similar to HMB, the skin lesions in HV are deep, with vesicles that are often hemorrhagic, leaving scars after healing. In contrast to HMB patients, many patients with HV do not show systemic symptoms even when the skin symptoms become aggravated. Patients with HV on rare occasions progress to show systemic symptoms and may be categorized under the diagnosis of CAEBV. In virtually all cases, monoclonal expansion of EBV is identified (23). As many patients with HMB show ectopic EBV infection of NK cells, patients with HV show activation and expansion of EBV-infected γδ T cells. Fractions of γδ T cells in peripheral blood are increased and are selectively activated, again offering tools for early diagnosis of the disease (24).

According to the data analyzed in Japan, age at onset >9 years old and activation of EBV-associated genes at the local skin lesions are regarded as poor prognostic factors (25). Furthermore, cases of benign classic-type illness progressing to aggressive systemic type illness have been reported (26). Monitoring the activity of EBV-infected T-cell clones by FCM and pathological analysis is therefore important.

## Flowcytometric analysis of circulating lymphocytes

FCM analysis of peripheral blood lymphocytes offers useful diagnostic information in some EBV-related diseases, and provides many advantages. FCM is a rapid assay requiring <1 h to complete. This analysis can be performed in any standard laboratory, and does not require a large amount of sample. Similar to other laboratory examinations, the assay can be repeated many times. For these reasons, FCM should be used as the first screening examination when EBV-associated T/NK-cell LPD is suspected. FCM examination consists of multiple steps.

First, we routinely examine the distributions of lymphocyte subpopulations as a screening assay to see if a particular subpopulation of lymphocytes is abnormally expanded. In many cases of EBV-associated T/NK-cell LPD, EBV-infected lymphocytes are increased in number. Simple analysis of the lymphocyte subpopulation will thus reveal abnormally increased NK cells, CD4^+^ T lymphocytes, CD8^+^ T lymphocytes or TCRγδ T cells. However, the increase in percentages of a particular lymphocyte subpopulation may vary depending on the clinical course, and further examination of the activation status of each lymphocyte subpopulation is therefore warranted.

**Figure 1:**
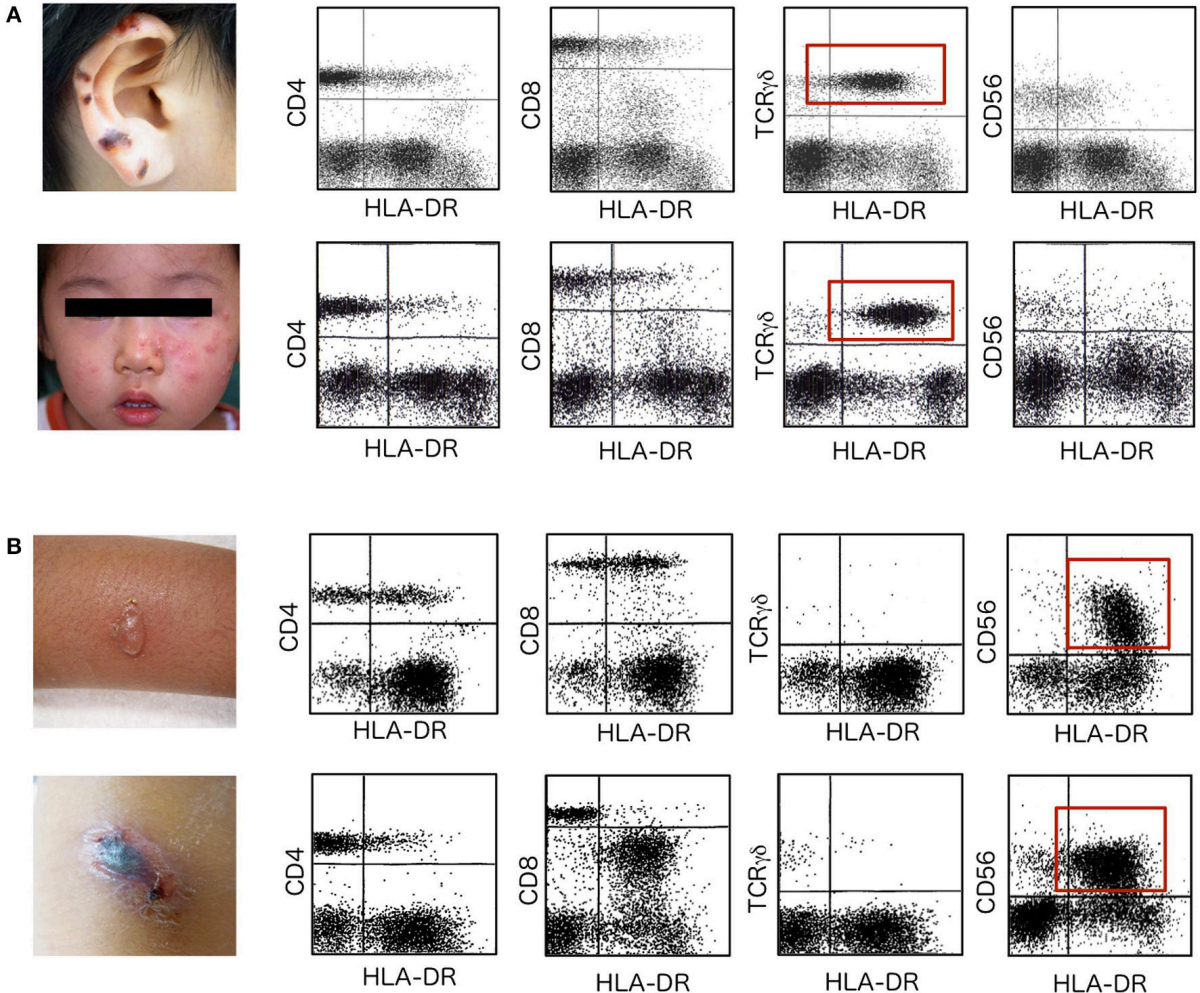
HLA-DR expression on lymphocyte subpopulations. Flowcytometric analysis of HLA-DR expression was performed to detect selective expansion and activation of a particular lymphocyte subpopulation in patients with HV and HMB. **(A)** Upper data are from a 12-year-old boy with hemorrhagic vesicles that started to appear 2 years earlier on sun-exposed areas of skin, including the earlobes. Lower data are from a 2-year-old girl who presented with clusters of erythematous vesicles on bilateral cheeks. In these two patients with HV, TCRγδ T cells are increased in number and express HLA-DR to significant levels (red squares). Other lymphocyte subpopulations, including CD4^+^ T cells, CD8^+^ T cells and CD56^+^ NK cells, express little HLA-DR on the cell surface. Skin lesions are frequently observed on UV-exposed areas including the ear lobes, cheek of the face and dorsal surfaces of the hands. **(B)** Upper data are from an 8-year-old girl who had a 5-year history of repeated episodes of vesicle formation and fever after mosquito bites. Lower data are from a 10-year-old boy who started to experience intense skin lesions with hemorrhagic vesicles with fever and general malaise 1 year before the first visit. In patients with HMB, CD56^+^ NK cells are increased and levels of HLA-DR expression are extremely increased (red squares).

In the next step, the activation status of each lymphocyte subpopulation is evaluated using expression of HLA-DR as an indicator of cell activation (Figure 1). Because clinical symptoms, including skin lesions, often suggest the clinical diagnosis and the target of EBV-infection, identifying selective activation of a particular lymphocyte subpopulation is particularly important when the responsible subpopulation is not increased to a significant level.

TCRγδ T cells are usually increased well above average levels in patients with HV (Figure 1A) (23). Furthermore, these cells are highly activated as suggested by the increased levels of HLA-DR expression. Activation is selective and no other lymphocyte subpopulations show increased HLA-DR in these cases. In contrast, NK cells are increased and highly activated in HMB (Figure 1B).

The third step is for cases with T-cell proliferation. We examine the distribution of TCR Vβ repertoire usages by FCM to identify any clonal expansion of either CD4^+^ or CD8^+^ T cells. In some cases, clonal expansion of EBV-infected T cells is recognized by commercially available monoclonal antibody (27). We can follow-up patients using the particular TCR Vβ as a sensitive marker for the presence of a sizable residual clone after treatment starts.

**Figure 2:**
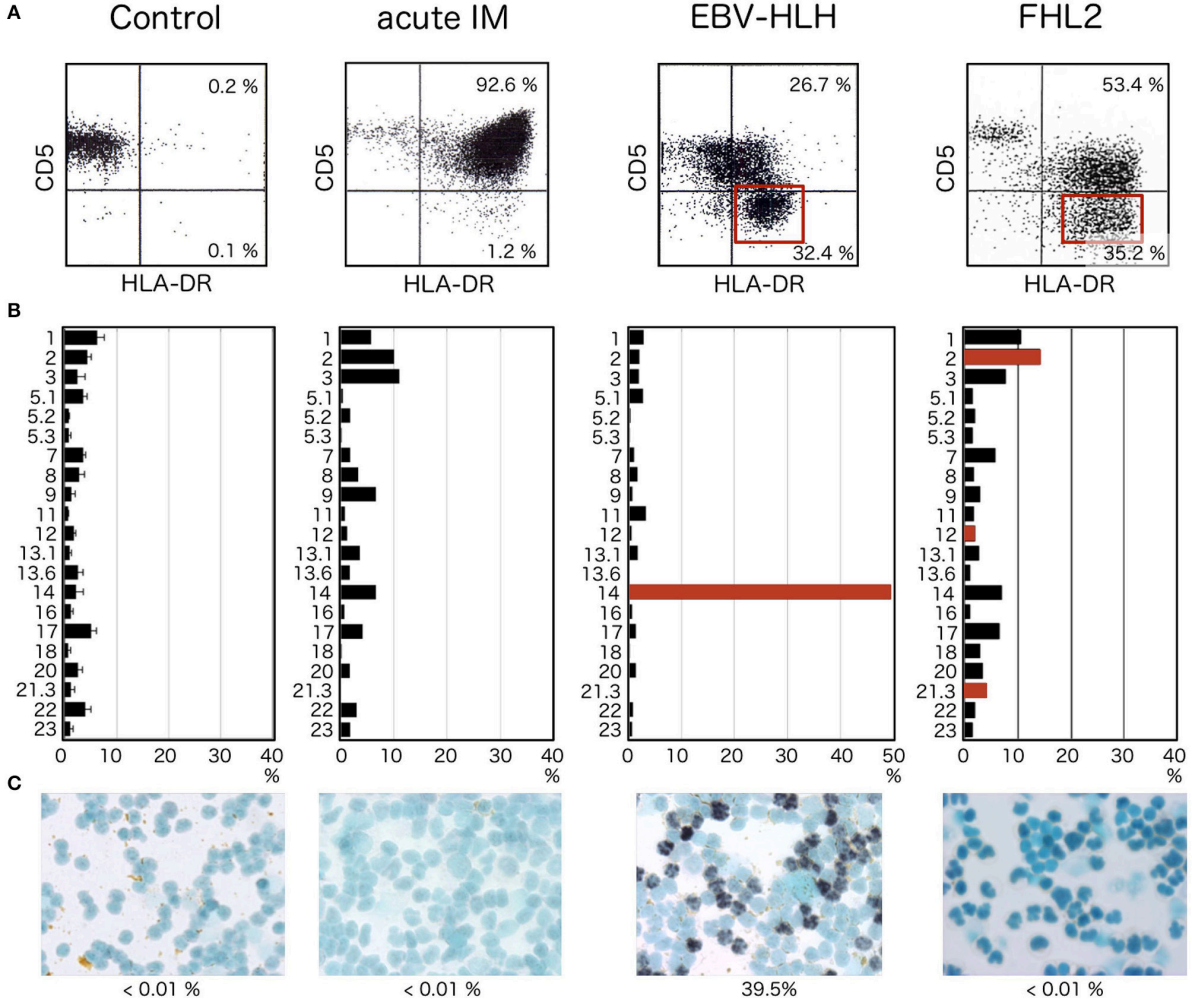
HLA-DR and CD5 expression in patients with acute HLH. **(A)** Expressions of CD5 and HLA-DR simultaneously examined by 3-color flowcytometry. Within the lymphocyte region, CD8^+^ cells are further gated and analyzed for expression of CD5 and HLA-DR. CD5^dim/negative^ HLA-DR^high^ CD8^+^ T cells are not detected in controls. Although CD8^+^ T cells express high levels of HLA-DR in acute infectious mononucleosis, most of these cells express normal, or only slightly decreased, levels of CD5. In contrast, significant fractions of CD5^dim/negative^ HLA-DR^high^ cells are seen within CD8^+^ T cells from EBV-HLH patients (red square). In cases with FHL, in which intense activation of oligoclonal T cells occurs as a response to EBV infection of B cells, significant reduction of CD5 is seen among CD8^+^ T cells (red square). **(B)** TCR Vβ distribution analyzed by FCM using commercially available monoclonal antibodies against different Vβ. Selective expansion of a single clone of CD8^+^ T cells is identified by a significant increase in T cells with a specific Vβ (red bar), whereas cells expressing other types of Vβ are universally suppressed. In patients with FHL2, several clones with different Vβs are activated with diminished expression of CD5 (red bars). **(C)** Only CD8^+^ T cells from EBV-HLH show EBER-1-positive cells within sorted CD8^+^ T-cell fractions. CD8^+^ T cells from controls or patients with acute infectious mononucleosis or FHL2 do not show EBER-1 positivity.

Finally, we look for a particular fraction of CD8^+^ T cells when EBV-HLH is suspected (Figure 2). For example, in acute IM, significant activation and expansion of CD8^+^ T cells is detectable. Increases in CD3^+^ T cells expressing high levels of HLA-DR and CD45RO^+^ memory CD8^+^ T cells are the characteristic findings in acute IM (28). It is important to note that the FCM findings of acute infectious mononucleosis and EBV-HLH are indistinguishable if only T-cell activation and increase in memory CD8^+^ T cells are analyzed. In the following paragraphs, examples of such analyses are shown.

In a series of studies comparing lymphocyte phenotypes in acute infectious mononucleosis and EBV-HLH, we found characteristic features observed only in EBV-HLH (Figure 2) (29, 30). Without exception, certain fractions among CD8^+^ T cells show lost or diminished expression of CD5 on the cell surface. These CD5^dim/negative^ CD8^+^ T cells express particularly significant levels of HLA-DR, forming distinct clusters of CD5^dim/negative^ HLA-DR^high^ cells among CD8^+^ T cells (Figure 2A, EBV-HLH, red square). These abnormal cell fractions can be detected at the earliest stage of EBV-HLH and offer a useful diagnostic clue. They are never detected in cases of acute IM, however severe the case is. In EBV-HLH, EBV infects a single clone of CD8^+^ T cells, leading to massive expansion and activation of the infected clone. Vigorous production of inflammatory cytokines, including interferon γ, by these activated cells is responsible for the significant clinical symptoms seen in EBV-HLH (31). Because the abnormal CD5^dim/negative^ HLA-DR^high^ CD8^+^ T cells are derived from a single clone, the clonal origin of these cells can be identified by analyzing expression profiles of TCR Vβ using FCM (Figure 2B, EBV-HLH). If the particular clone uses TCR Vβ detectable by commercially available antibody, selective expansion of cells with a particular TCR Vβ can be identified as CD5^dim/negative^ HLA-DR^high^ CD8^+^ T cells.

Percentages of CD5^dim/negative^ HLA-DR^high^ CD8^+^ T cells correlate relatively well with serum ferritin levels and EBV copy numbers in peripheral blood. Detection of this particular cell fraction is thus useful not only for early diagnosis of EBV-HLH, but also valuable for easy, repeatable evaluation of the clinical responses to therapy and prediction of the prognosis. Percentages of the abnormal cell fraction reduce rapidly in parallel with response to appropriate therapy, improvement of clinical symptoms and normalization of laboratory data, including ferritin and soluble interleukin-2 receptor.

Although downregulation of CD5 antigen within the CD8^+^ T-cell fraction is a characteristic finding in cases of acute EBV-HLH and facilitates early diagnosis, the finding is not necessarily specific to EBV-HLH. FCM analysis alone is insufficient to make a definitive diagnosis unless the CD5^dim/negative^ fraction is determined to be of a single clone by TCR Vβ repertoire analysis. We have encountered multiple cases of familial hemophagocytic lymphohistiocytosis (FHL), in which HLA-DR^high^ CD5^dim/negative^ fractions can be identified within CD8^+^ T cells (Figure 2A, FHL2, red square). The target of EBV infection is the B cell in patients with FHL, but activation and expansion of oligoclonal CD8^+^ T cells are associated with downregulation of CD5 for unknown reason (32, 33). Confirming that the target of EBV infection is CD8^+^ T cells and not B cells is therefore important whenever patients with an increased EBV copy number and HLH phenotype show oligoclonal expansion of HLA-DR^high^ CD5^dim/negative^ cells (Figure 2B). CD8^+^ T cells are the target of EBV infection only in cases with EBV-HLH (Figure 2C). No CD8^+^ T cell is infected with EBV in acute IM or FHL cases.

Since our publications of the first article on EBV-HLH and CD5 expression (29, 30), we have experienced more than 50 cases of acute EBV-HLH, later confirmed by EBV clonality and the ectopic EBV infection to CD8^+^ T cells. Without exception, we could identify the unique CD5^dim/negative^ HLA-DR^high^ CD8^+^ T cells in these cases. The percentages of this population within CD8^+^ T cells ranged from 5 to 90%. It is important to note that one should focus on large cells to detect the abnormal cells and bone marrow may serve as a better source for the analysis (unpublished data). Clonal expansion of CD8^+^ T cells may not be identified by available monoclonal antibodies. Our experience is that in 50–60% of the cases, the abnormal expansion of CD8^+^ T cells with particular TCR Vβ repertoire can be identified. When clonal expansion is not confirmed by flow cytometry, it is necessary to separate lymphocyte subpopulations and identify the target of EBV infection by either EBER-1 *in situ* hybridization or real time PCR. Whenever EBV infection was found in B cells, and not in CD8^+^ T cells, one should suspect FHL and genetic analysis should be required.

## Conclusion

Once suspected, EBV-associated T/NK-cell LPD is not difficult to diagnose and appropriate treatment can be initiated with successful control of the disease process. However, large numbers of patients remain undiagnosed or lose their life without suspicion of the disease. It is the responsibility of the physician to suspect the possibility of EBV-associated T/NK-cell LPD at their early stages. FCM analysis at different levels offers rapid and often precise diagnostic information to reach the final diagnosis of EBV-HLH or CAEBV. In combination with more tedious procedures for the identification of EBV-infected lymphocyte subpopulations, FCM data are useful in the initial process of diagnosing EBV-associated T/NK-cell LPD.

## Author contributions

AY obtained and analyzed the data, and organized the final structure of the manuscript.

### Conflict of interest statement

The author declares that the research was conducted in the absence of any commercial or financial relationships that could be construed as a potential conflict of interest.
